# Papillary fibroelastoma revealed by an acute coronary syndrome with transient ST segment elevation: a case report

**DOI:** 10.11604/pamj.2022.41.206.33077

**Published:** 2022-03-14

**Authors:** Saïd Makani, Amal Haoudar, Abderahmane Al Bouzidi, Chafik Elkettani, Mahdi Ait Houssa

**Affiliations:** 1 Cardiac Surgery Department, Mohammed VI University of Health Sciences, Casablanca, Morocco,; 2 Cardiac Anesthesia and Intensive Care Department, Mohammed VI University of Health Sciences, Casablanca, Morocco,; 3 Pathological Anatomy Department, Mohammed VI University of Health Sciences, Casablanca, Morocco

**Keywords:** Heart neoplasms, coronary disease, papillary fibroelastoma, acute coronary syndrome, case report

## Abstract

Cardiac papillary fibroelastoma is a rare, benign tumour, arising from the valvular endocardium, which could lead to life-threatening complications as myocardial ischemia. We report a case of a 54-year-old male patient who presented in the emergency room with an acute coronary syndrome with transient ST segment elevation. After ruling out coronary artery disease by coronarography, we established the diagnosis of papillary fibroelastoma by performing echocardiogram completed by computed tomography angiography. The reversible acute coronary syndrome has been caused by the prolapse of pedunculated coronary cusp tumour into the main left coronary ostium. The patient was scheduled for emergent surgery. The surgical management included a complete resection of the tumour sparing the aortic valve. The patient recovered well. A papillary fibroelastoma of the aortic valve can be revealed by an acute coronary syndrome with transient ST segment elevation. More investigations must be done to eliminate such diagnosis in the case of a normal coronarography.

## Introduction

Cardiac papillary fibroelastoma is a rare tumour, histologically benign, mostly attacks heart valves, predominantly the aortic valve, following by the mitral valve. It affects adults between the fourth and eighth decade of life, with an incidence of 0.02% [1,2]. While most patients are asymptomatic and the tumour were incidentally diagnosed, others may have life threatening complications like myocardial infarction or stroke [3]. We report a case of a 54-year-old male patient presented in our emergency room with an acute coronary syndrome, the coronarography shows no lesions. The diagnosis was a papillary fibroelastoma of the aortic valve which had a flap effect occluding the main left coronary artery.

## Patient and observation

**Patient information:** a 54 year-old-male patient, with no risk factors and without medical history, who was admitted to the emergency room for chest pain and dyspnea.

**Figure 1:**
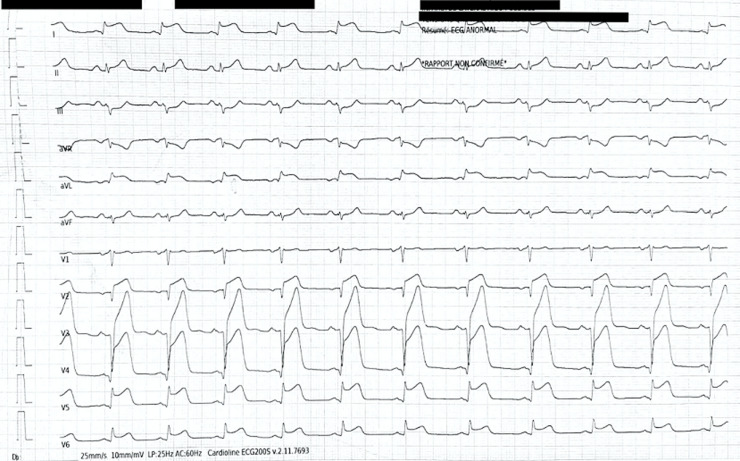
an electrocardiogram shows an ST segment elevation in the anterior leads

**Clinical findings:** the physical examination was normal. Despite the patient´s stable clinical condition, the electrocardiogram showed a transient ST segment elevation in the anterior leads (Figure 1).

**Timeline of the current episode:** 13 March 2021 at 9 am, the patient felt acute chest pain, at 9:30 am the patient was admitted to the emergency room, at 11 am the coronarography was performed, the echocardiogram and the CT scan was realized during the same day. 14 March 2021 at 8:30 am the patient was admitted to the operating room. 20 march 2021 the patient was discharged from the hospital.

**Figure 2:**
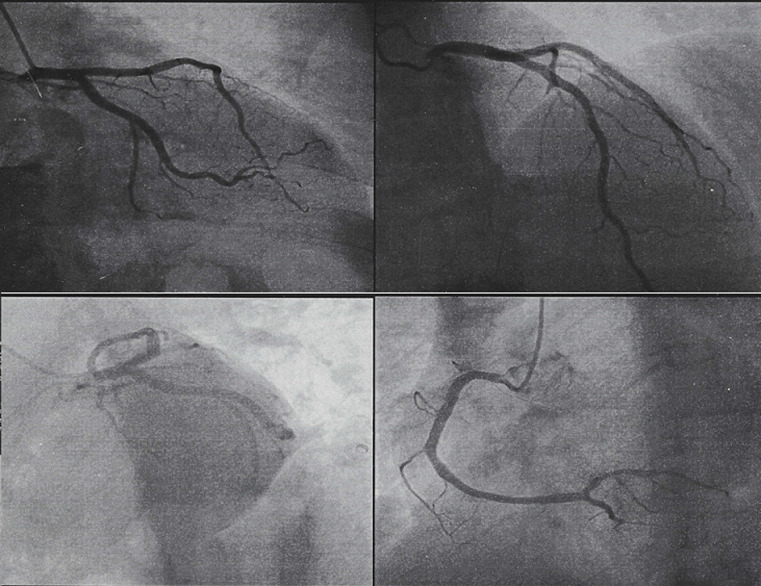
a coronarography realized shows no lesions

**Figure 3:**
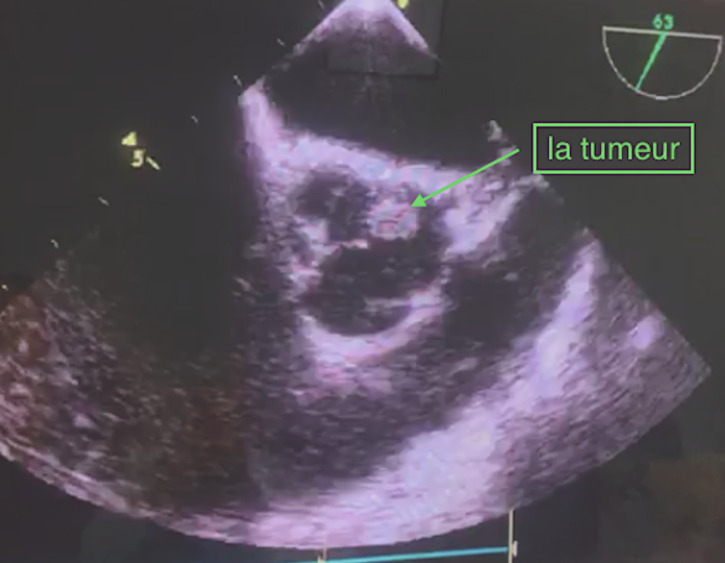
transoesophageal echocardiography short axis view, arrow points to a 10mm /8mm tumour, attached to the non/left commissure

**Figure 4:**
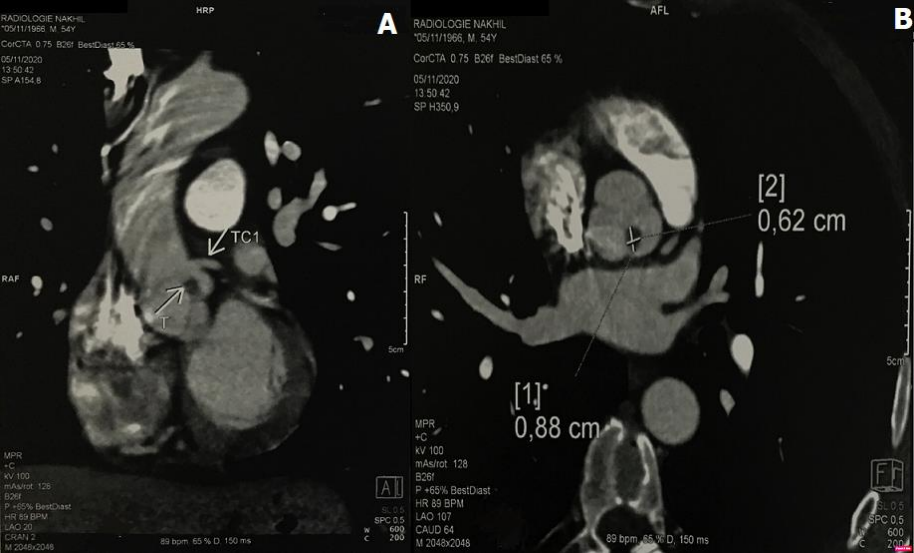
computed tomography angiography shows a 0.62/0.88 cm mass attached to aortic leaflet, near the ostium of the main left coronary artery (A, B)

**Diagnostic assessment:** an urgent coronarography was performed in the catheter laboratory, which was normal (Figure 2). In the echocardiogram, the cardiologist suspects active endocarditis of the aortic valve, we complete with a transoesophageal echocardiogram which reveals the existence of a pedunculated rounded structure attached to the aortic valve, no aortic stenosis or regurgitation, and moderate left ventricle dysfunction (Figure 3). A computed tomography angiography shows a 8.8mm/6.2mm bell-stick mass, attached to the non/left commissure of the aortic valve (Figure 4).

**Diagnosis:** the presumptive diagnosis consisted of a tumour of the aortic valve, the nature of the tumour will be revealed by the histopathology of the excised mass.

**Figure 5:**
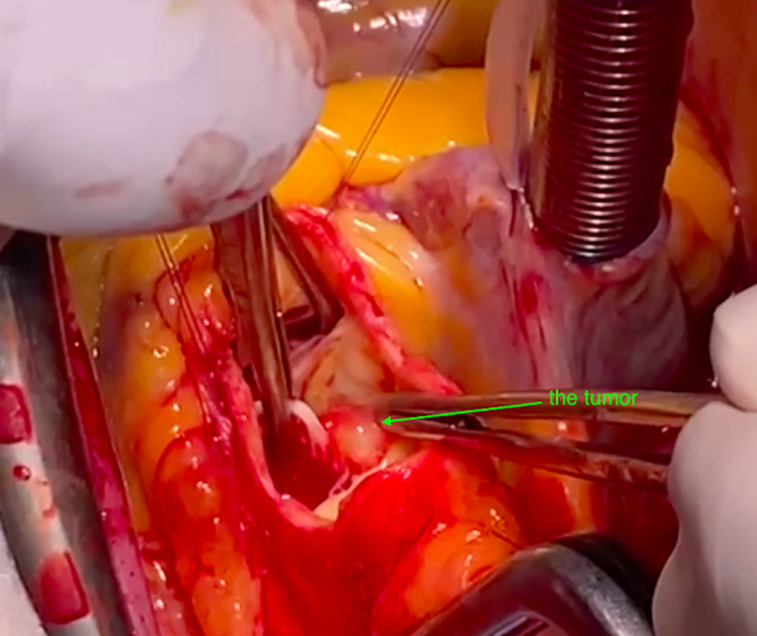
intraoperative view, arrow points to the mass in the left/ non commissure of the aortic valve

**Figure 6:**
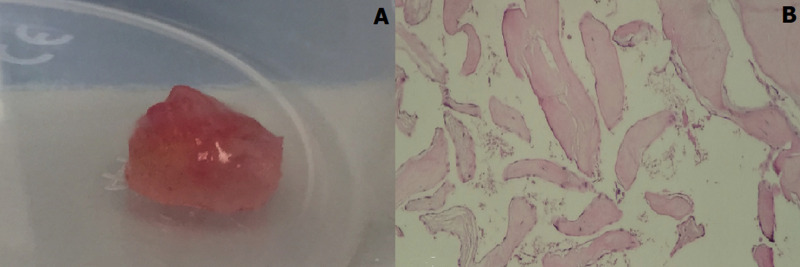
macroscopic aspect of the excised mass (A) and histological section of the excised mass, papillae bordered by regular endothelial cells resting on a hyalinized connective axis consistent with papillary fibroelastoma (HE, G x100) (B)

**Therapeutic interventions:** the patient was scheduled for emergency surgery. He underwent a median sternotomy and was placed on cardiopulmonary bypass. The aorta was crossclamped and an oblique aortotomy was performed as usual. The patient underwent excision of the tumour which was attached to the non/left commissure of the aortic valve and were approximately 1 cm in diameter. Valve leaflets were preserved and no other lesion was discovered. The coronary ostia were normal (Figure 5, Figure 6A). Histopathologic results were consistent with papillary fibroelastoma of the aortic valve, without signs of malignancy (Figure 6B).

**Follow-up and outcome of interventions:** the patient´s postoperative course was uneventful, and he was doing well at his 9 months follow-up examination.

**Patient perspective:** “when I had chest pain I was very scared, especially when I heard that I had a tumour, then I was relieved by the surgeon, who told me that was a benign tumour with a low rate of recurrence. I feel a total recovery and I continue with clinical follow-up”.

**Informed consent:** ethical approval was obtained from the director of the Hospital. Formal approval is not necessary to report a case as “case report” in our hospital. The written consent for publication was released by the patient.

## Discussion

Here we report an uncommon case of a patient who survived an acute coronary syndrome, the papillary fibroelastoma had a flap effect occluding the left main coronary artery but the specificity of our case that the obstruction was transient, there was neither cardiac arrest nor myocardial infarction, the patient´s cardiac enzyme levels were normal. Ikegami H *et al* reported a case of cardiac arrest from the left main coronary artery ostial obstruction, caused by a cardiac papillary fibroelastoma, the patient presented with severe coronary ischemia and progressed to cardiac arrest, for which she was resuscitated [4].

Primary cardiac tumours are a rare disease, papillary fibroelastoma comes second after myxoma with an incidence of 10% of all benign cardiac tumours [5] and the most common primary tumour of heart valves, chiefly the aortic valve; the mitral, tricuspid, and pulmonary valves are less often involved [6]. It is usually asymptomatic and the diagnosis is mostly incidental, some patients may experience embolic complications such as stroke, acute coronary syndrome or myocardial infarction, which could lead to death from coronary ostial obstruction [7] which is may be caused by the embolization of the thrombus produced on the tumour surface or of the tumour itself.

Echocardiography, as a first line exam, is useful in identifying the tumour and a possible valve dysfunction [8], the diagnosis can be done easily with computerized tomography (CT) and magnetic resonance imaging (MRI) [9]. In the management of papillary fibroelastoma, treatment strategies are controversially discussed, oral anticoagulation can be a therapeutic option if the tumour is small < 1cm and not mobile or if valve replacement is strongly expected at the time of tumour resection. Gowda and colleagues reported tumour-related death in 12 of 25 patients medically treated [10]. Surgery is the best therapeutic option for primary cardiac tumours with excellent results [1], it´s indicated relatively urgently and recommended in symptomatic as well as asymptomatic patients because of the risk of embolization, especially when the tumour is > 1cm [4]. Although the natural history of cardiac papillary fibroelastoma is largely unknown, the successful complete resection of the papillary fibroelastoma is curative and the long-term postoperative prognosis is excellent, in the literature, the recurrence rate is lower than 2% [2,4].

## Conclusion

Patients with angina that are not explained by coronary lesions must have an echocardiography to exclude embolic sources. Although rare and benign, cardiac papillary fibroelastoma can lead to life-threatening complications, acute coronary syndrome, sudden death or stroke, the possibility of this diagnosis should be kept in mind while managing cardiac or valvular tumours, it requires a strong suspicion and appropriate use of imaging modality. The optimal surgical procedure is valve-sparing resection. Although tumoral regrowth is rare after surgical excision, long-term follow-up is essential.
